# Effects of Stop‐Work orders on HIV testing, treatment and programmes for prevention of vertical transmission in four sub‐Saharan African countries

**DOI:** 10.1002/jia2.70034

**Published:** 2025-09-04

**Authors:** Suzue Saito, Mansoor Farahani, Salaza Kunda, Lievain Maluantesa, Agnaldo Guambe, Habtamu Ayalneh Worku, Eugenie Poirot, Nyikadzino Mahachi, Lucille Bonaventure, Stéphania Koblavi, Tafadzwa Dzinamarira, Wafaa M. El‐Sadr

**Affiliations:** ^1^ ICAP at Columbia University New York New York USA; ^2^ ICAP at Columbia University Lusaka Zambia; ^3^ ICAP at Columbia University Kinshasa Democratic Republic of the Congo; ^4^ ICAP at Columbia University Luanda Angola; ^5^ ICAP at Columbia University Juba South Sudan

**Keywords:** antiretroviral therapy, HIV testing, PEPFAR, service disruption, Stop‐Work orders, sub‐Saharan Africa

## Abstract

**Introduction:**

Beginning in late January 2025, Stop‐Work orders and contract cancellations have disrupted HIV programmes supported by the President's Emergency Plan for AIDS Relief (PEPFAR). We assessed the effects on HIV service delivery in four African countries.

**Methods:**

Weekly aggregate HIV services data from a convenience sample of 165 Center for Disease Control and Prevention (CDC)‐funded, ICAP‐supported facilities—22 in Angola, 75 in the Democratic Republic of the Congo (DRC), 20 in South Sudan and 48 in Zambia—were analysed. We compared data from pre‐Stop‐Work (7 October 2024–23 January 2025), Stop‐Work (24 January 2025–11 February 2025) and post‐resumption (12 February 2025–31 March 2025) phases. We examined the number of individuals: (1) who tested for HIV; (2) receiving index testing; (3) had HIV‐positive results/yield; (4) initiated antiretroviral therapy (ART); as well as (5) number of pregnant women with known HIV status; and (6) number of HIV‐exposed infants who received early infant diagnosis (EID) testing. We used phase‐specific weekly averages, relative percentage changes across phases and linear trend tests to measure the magnitude of disruptions and recovery.

**Results:**

In Angola, DRC and Zambia, significant declines in number of HIV‐positive tests (−58%, −34%, −17%) and ART initiations (−16%, −32%, −17%) were observed across the three phases with limited recovery in number of positive tests in Zambia and ART initiations in Angola. In DRC and Zambia, HIV testing (−33%, −35%), including index testing (−37%, −72%), significantly declined; additionally, HIV testing of pregnant women significantly declined (−28%) in DRC. In Angola and Zambia, EID testing declined (−12%, −18%) with limited recovery. In Angola, HIV testing (2476→2205→2519), including testing for pregnant women (280→ 233→ 287), rebounded in the post‐resumption phase; in DRC, EID (6.5→6.3→7.9) rebounded. There were increases in HIV testing yield in Zambia (2.8%→3.1%→4.0%) and index testing (20→24→36) in Angola. No reductions were observed in South Sudan.

**Conclusions:**

Stop‐Work orders and award terminations have resulted in substantial short‐term reductions in the delivery of HIV testing and treatment services. Long‐term funding disruptions necessitate careful planning, realistic timelines and investment in cost‐effective service models to sustain the gains and maintain the momentum in the global HIV response.

## 1 INTRODUCTION

On 20 January 2025, an executive order entitled “Re‐evaluating and Re‐aligning United States (US) Foreign Aid” paused all foreign development assistance for 90 days to assess programmatic efficiencies and policy consistency [1]. The order immediately halted all programmes funded by the President's Emergency Plan for AIDS Relief (PEPFAR) through Stop‐Work orders issued by the United States Agency for International Development (USAID) and the Centers for Disease Control and Prevention (CDC), the two agencies supporting the vast majority of PEPFAR programming. On 11 February, PEPFAR/CDC‐funded programmes were permitted to resume under a temporary restraining order. Most PEPFAR/USAID‐funded programmes received terminations; a few programmes that remained operated under a limited waiver for HIV testing, treatment and prevention of vertical transmission (PVT) activities issued by the Bureau for Global Health Security and Diplomacy of the State Department. The uncertainty of continued funding has compelled ministries of health and implementing partners to scale back activities and retrench staff, even for those programmes that had been permitted to continue the work. In this context, news reports described HIV patients being turned away from clinics without receiving their antiretroviral medicines [2] and the consequences of interruption of treatment [3].

Disruptions in HIV testing services can cause delays in diagnosis for people living with HIV (PLH), while disruptions in the initiation or continuation of antiretroviral therapy (ART) not only threaten the health of PLH but also increase the risk of spread of the virus, including vertical transmission from mothers to infants. One modelling study predicted that discontinued PEPFAR support across low‐ and middle‐income countries could result globally in an additional 4.43–10.75 million new HIV acquisitions and 0.77–2.93 million HIV‐related deaths between 2025 and 2030 compared to the status quo [4]. Another modelling study showed that withdrawal of such support would result in an additional 1 million children acquiring HIV, 0.5 million additional children will die of AIDS and 2.8 million children will additionally become orphaned by AIDS by 2030 [5].

In this brief report, we measured the trends in HIV testing, treatment initiation and PVT services at a subset of health facilities funded by PEPFAR/CDC and supported by ICAP in four countries—Angola, the Democratic Republic of Congo (DRC), South Sudan and Zambia—as a consequence of the Stop‐Work orders. Additionally, PEPFAR/USAID community‐based testing programmes referred patients to PEPFAR/CDC facility‐based treatment programmes in Angola, DRC and Zambia. In South Sudan, there were no PEPFAR/USAID community‐based testing programmes referring to facilities that ICAP worked.

## 2 METHODS

### 2.1 Study design and context

This study employed a retrospective analysis of routinely collected health services data from 165 health facilities funded by PEPFAR/CDC and supported by ICAP, including 22 in 4/18 provinces in Angola, 75 in 1/26 provinces in the DRC, 20 in 2/10 states in South Sudan and 48 in 2/10 provinces in Zambia, to assess the effects of operational disruptions on HIV testing, treatment initiation and PVT services. This was a convenience sample of facilities that provided HIV treatment to 12.2%, 3.3%, 30.8% and 4.5% of the total number of PLH in Angola, DRC, South Sudan and Zambia, respectively, according to the latest national numbers. All study health facilities offered Ministry of Health ‐approved HIV treatment services. The analysis period encompassed early October 2024 through late March 2025, spanning three distinct phases: pre‐Stop‐Work (early October 2024 through 23 January 2025), Stop‐Work (24 January 2025, through 11 February 2025) and post‐resumption (12 February 2025, through 31 March 2025). This timeframe provided an adequate pre‐disruption baseline and sufficient post‐resumption follow‐up to observe initial recovery patterns.

### 2.2 Data sources and collection

Weekly aggregated health services data were abstracted from paper‐based or electronic data capture systems at PEPFAR/CDC‐funded, ICAP‐supported facilities in each country. These data represent routine programmatic performance metrics and are tracked through established health information systems.

### 2.3 Key indicators

Six HIV indicators were selected to assess the effects of Stop‐Work orders: (1) total number of HIV tests conducted across different types of HIV testing services; (2) the number of contacts of HIV‐positive individuals tested for HIV (index testing); (3) the number of individuals testing HIV positive; (4) the number of new HIV patients initiated on ART; (5) the number of pregnant women with known HIV status, either through HIV testing or prior diagnosis in PVT programmes; and (6) the number of HIV‐exposed infants who had early infant diagnosis (EID) testing. Additionally, HIV‐testing yield was calculated by dividing the number of HIV‐positive tests by the total number of HIV tests performed to assess the efficiency of testing approaches. These indicators were selected because they would have been among the services stopped during the Stop‐Work order phase and resumed under the waiver. Note that data were unavailable for the number of pregnant women with known HIV status and for the number of HIV‐exposed infants who underwent EID from South Sudan at the time of writing this report. We additionally collected facility information on ownership (private/public), residence (urban/rural), facility type (hospital, health centre/post) and the number of PLH active on HIV treatment as of December 2024.

### 2.4 Analytical approach

For each country and indicator, we calculated the weekly average number of patients receiving services within each phase (pre‐Stop‐Work, Stop‐Work and post‐resumption) to facilitate comparison across phases. Relative percentage changes were computed between weekly average counts measured in pre‐Stop‐Work and Stop‐Work phases to summarize the immediate impact of service disruptions. Similar calculations were performed to assess recovery trends by comparing post‐resumption averages with pre‐Stop‐Work baselines. To distinguish routine operational variation from meaningful change, we examined the distribution of absolute adjacent‐week percent changes during the pre‐Stop‐Work phase; 95% of changes fell within ±10% across indicators and countries. We summarized overall trends across the three phases as follows: “<10% change” for fluctuations within ±10% (representing normal weekly variation for these data, as 95% of absolute adjacent‐week changes in the pre‐Stop‐Work phase fell within ±10%); “decline” for trends showing ≥10% decreases across phases; “recovery” for trends showing ≥10% declines during the Stop‐Work phase followed by improvement post‐resumption; and “increase” for trends showing a ≥10% increase across phases. For the DRC, where pre‐Stop‐Work data were monthly rather than weekly, we evaluated adjacent‐month percent changes and applied the same classification rule. We conducted complete case analyses. Overall, we were missing 1.6% (311/19,875) of weekly count data, ranging from 0.04% in DRC to 4.5% in Zambia.

Descriptive statistics were calculated using Stata 18 (StataCorp, College Station, TX, USA). We additionally ran ordinary least squares linear regression on weekly counts to determine statistically significant trends. Analyses of these routine health services data have received non‐research determinations from the CDC (2017‐519).

## 3 RESULTS

A total of 165 facilities were included in the analysis across the four countries. The majority of the facilities were public facilities: 94% in Angola, 55% in DRC, 91% in Zambia and 100% in South Sudan. The remaining were faith‐based or private facilities. These facilities were largely situated in urban areas, in Angola (95.5%), DRC (59.0%) and Zambia (54.2%), while facilities in South Sudan were mainly (60%) in rural locations. The majority were health centres, in all four countries: 50% in Angola, 85% in DRC, 60% in South Sudan and 61% in Zambia. The average number of PLH receiving HIV treatment per facility ranged from 191 in DRC, 889 in Angola, 969 in South Sudan, to 1239 in Zambia.

**Figure 1:**
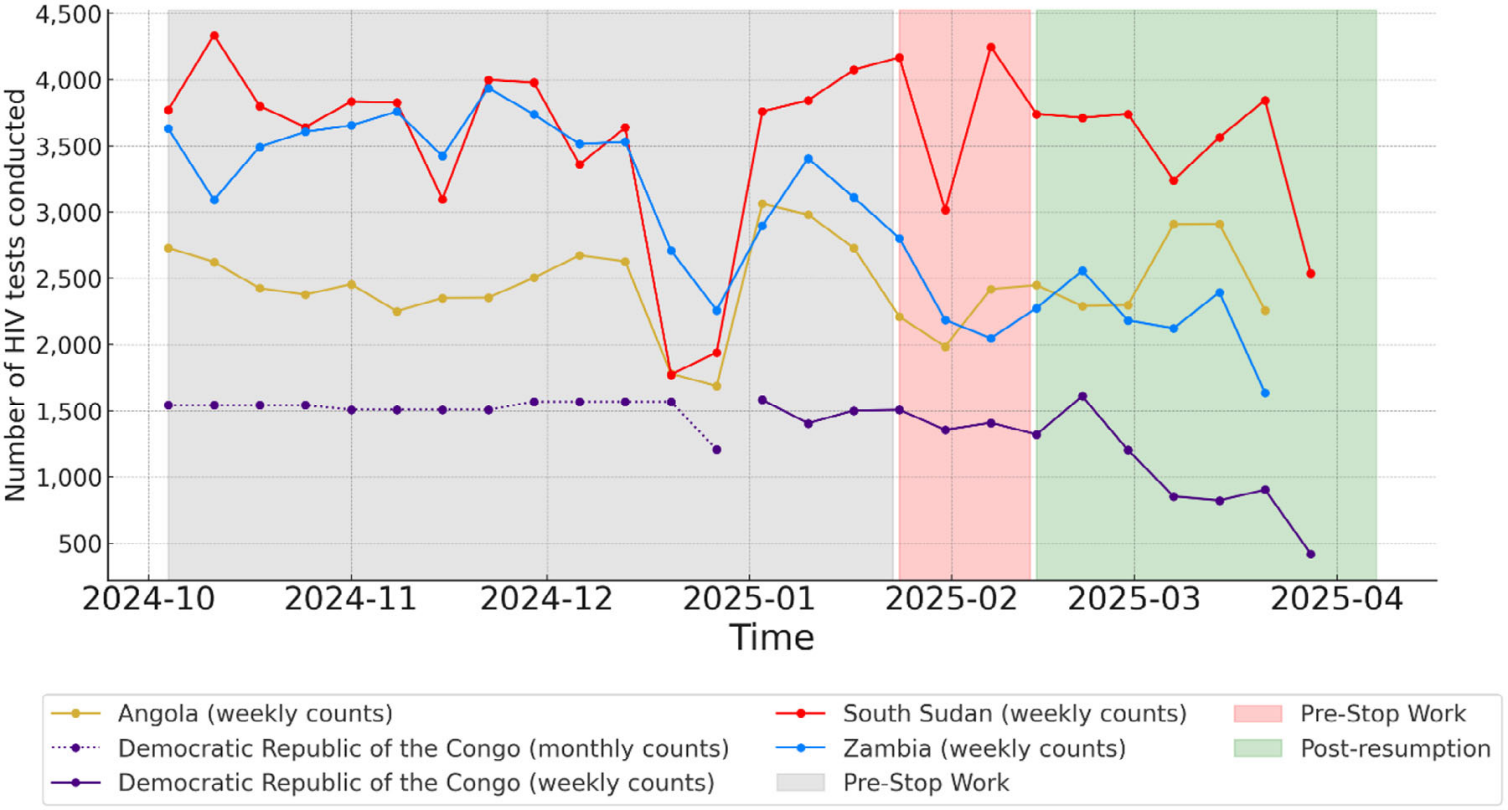
Number of HIV tests in Angola, DRC, South Sudan and Zambia, October 2024−March 2025.

Table 1 summarizes the weekly average counts of the selected indicators in each country across the three phases of work. In Angola, the average number of HIV tests decreased during the Stop‐Work phase and rebounded to baseline levels in the post‐resumption phase. In South Sudan, HIV testing remained stable with fluctuations within 10% across the three phases. In DRC and Zambia, HIV testing declined during the Stop‐Work phase and continued to decline without recovery during the post‐resumption phase (*p*<0.05) (Figure 1 and Table 1).

**Table 1:** Effects of Stop‐Work orders on various HIV service indicators and recovery patterns in four sub‐Saharan African countries (October 2024–March 2025)

HIV indicator	Country	Pre‐Stop‐Work phase (weekly mean)	Stop‐Work phase (weekly mean)	Post‐resumption phase (weekly mean)	% Change (Stop‐Work vs. pre‐Stop‐Work phase)	% Change (post‐resumption vs. pre‐Stop‐Work phase)	Trend across pre‐Stop‐Work, Stop‐Work and post‐resumption phases^*^
**Number of HIV tests**	**Angola**	2475.8	2204.7	2519.3	−11.0%	1.8%	Recovery
	**DRC**	1511.3	1424.3	1019.3	−5.8%	−32.6%	Decrease^*^
	**South Sudan**	3542	3811	3482.9	7.6%	−1.7%	<10% change
	**Zambia**	3359.9	2345	2194.8	−30.2%	−34.7%	Decrease^*^
**Number** **of HIV‐positive tests**	**Angola**	114.2	87	47.5	−23.8%	−58.4%	Decrease^*^
	**DRC**	31.2	30.3	20.7	−2.7%	−33.6%	Decrease^*^
	**South Sudan**	49.9	48.3	48.6	−3.1%	−2.6%	<10% change
	**Zambia**	92	73.7	76	−19.9%	−17.4%	Decrease^*^/Recovery
**HIV positivity yield**	**Angola**	4.6	3.9	1.9	−14.4%	−57.9%	Decrease^*^
	**DRC**	2	2.1	1.7	3.2%	−15.4%	Decrease^*^
	**South Sudan**	1.4	1.3	1.4	−8.0%	0.9%	<10% change
	**Zambia**	2.8	3.1	4	13.4%	44.1%	Increase^*^
**Number of HIV index tests**	**Angola**	20.4	23.7	35.7	16.2%	75.1%	Increase^*^
	**DRC**	27.5	12.3	17.4	−55.2%	−36.6%	Decrease^*^/Recovery
	**South Sudan**	111.8	111.3	108.6	−0.4%	−2.9%	<10% change
	**Zambia**	277.2	110	78.5	−60.3%	−71.7%	Decrease^*^
**Number of new HIV treatment initiations**	**Angola**	49.8	38.7	41.8	−22.4%	−16.0%	Decrease^*^/Recovery
	**DRC**	31.1	29	21.3	−6.6%	−31.5%	Decrease^*^
	**South Sudan**	52	51	48	−1.9%	−7.7%	<10% change
	**Zambia**	89.2	78.3	74.2	−12.2%	−16.9%	Decrease^*^
**Number of pregnant women with known status**	**Angola**	279.3	232.7	286.7	−16.7%	2.6%	Recovery
	**DRC**	741.2	727.7	533.4	−1.8%	−28.0%	Decrease^*^
	**Zambia**	400.3	440.3	423	10.0%	5.7%	<10% change
**Number of infants who received early infant diagnosis**	**Angola**	37.9	30.7	33.2	−19.0%	−12.4%	Decrease^*^/Recovery
	**DRC**	6.5	6.3	7.9	−2.6%	20.9%	Recovery
	**Zambia**	71.2	57	58.5	−19.9%	−17.8%	Decrease^*^/Recovery

* Linear regression trend statistic *p*<0.05.

Regarding the number of HIV‐positive tests, the average weekly number showed a decreasing trend across the time phases in both Angola and the DRC (*p*<0.05). In South Sudan, it remained stable, fluctuating within a 10% range. In Zambia, the number of HIV‐positive tests declined during the Stop‐Work phase (*p*<0.05), recovering partially in the post‐resumption phase. For index testing, in Angola, the weekly average number of index tests increased over the three phases (*p*<0.05). In DRC, index testing declined during the Stop‐Work phase (*p*<0.05) and partially recovered, while in South Sudan, it remained stable. Lastly, in Zambia, index testing substantially declined during the Stop‐Work phase and continued to decline (*p*<0.05), albeit at a slower rate, during the post‐resumption phase.

The yield for HIV testing declined in Angola across the three phases (*p*<0.05). In DRC, the yield remained stable during the Stop‐Work phase before declining during the post‐resumption phase (*p*<0.05), while it remained stable in South Sudan. In Zambia, the yield increased across the three phases (*p*<0.05).

**Figure 2:**
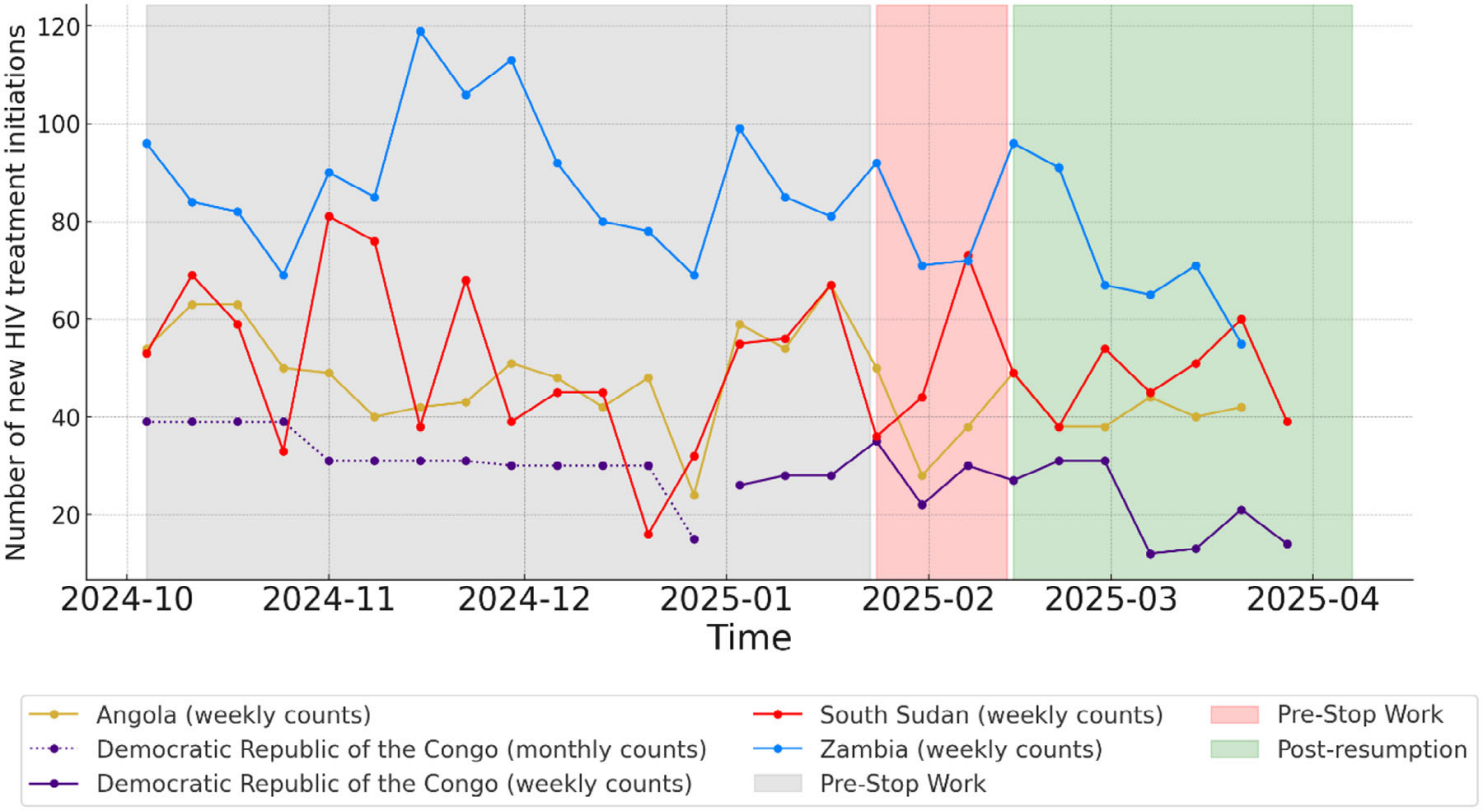
Number of new HIV treatment initiations in Angola, DRC, South Sudan and Zambia, October 2024−March 2025.

HIV treatment initiation declined during the Stop‐Work phase in Angola (*p*<0.05), with limited recovery during the post‐resumption phase. In South Sudan, HIV treatment initiation was relatively stable, although initiations slightly declined across the three phases. In DRC and Zambia, new HIV treatment initiations decreased across the three phases (*p*<0.05) (Figure 2 and Table 1).

With regard to PVT services, in Angola, the number of pregnant women with known HIV status declined during the Stop‐Work phase but rebounded during the post‐resumption phase to baseline levels. In DRC, the number decreased across the phases, particularly in the post‐resumption phase (*p*<0.05), while in Zambia, the number was relatively stable. The number of HIV‐exposed infants who received EID in Angola and Zambia significantly decreased during the Stop‐Work phase (*p*<0.05) with limited recovery; in DRC, EID rebounded during the post‐resumption phase.

## 4 DISCUSSION

In this report, we present what we believe to be one of the first data on the effects of the Stop‐Work Orders on the delivery of HIV testing, treatment and PVT services in sub‐Saharan Africa using facility‐level HIV services data [6]. We observed varying effects on HIV services across indicators and countries. In Angola, DRC and Zambia, significant declines in the number of HIV‐positive tests and ART initiations were observed even after the resumption of work, with limited recovery in ART initiations in Angola and a number of positive tests in Zambia. In DRC and Zambia, HIV testing, including index testing, as well as HIV testing in pregnant women in DRC, significantly declined across the three phases. In Angola and Zambia, EID testing declined substantially, with limited recovery in the post‐resumption phase. The continued reductions in services were due to several factors, including terminations of community‐based HIV testing services and commodity distributions funded by PEPFAR/USAID, contributing to the reduction in treatment numbers in the post‐resumption phase in Angola, the DRC and Zambia. In the DRC, additionally experienced widespread lockdowns due to the takeover of Eastern regions of the country by rebels, which likely contributed to part of the reductions. In contrast, health facilities in South Sudan did not experience prolonged disruptions due in part to the absence of PEPFAR/USAID‐supported HIV programming that integrated with PEPFAR/CDC‐funded programmes in the regions where ICAP supports HIV services. Additionally, pre‐positioned buffer stock of commodities in anticipation of potential unrest as a consequence of the presidential election also contributed to pre‐empting any shortages as a result of Stop‐Work orders.

In contrast, in Angola, HIV testing, including testing for pregnant women, rebounded in the post‐resumption phase, owing to a quick remobilization of staffing resources. Further, there were increases in HIV testing yield in Zambia and index testing in Angola. In Zambia, the increase in testing yield, despite a decrease in HIV tests performed, is likely due to the fact that during the Stop‐Work phase, low‐yield HIV testing approaches, such as retesting for breastfeeding women, testing of women attending family planning services and community‐based testing, were scaled back. Yet, new HIV treatment initiations were continued to decline in Zambia, suggesting the difficulties in restarting HIV treatment services after even a brief disruption. In Angola, the increase in index testing is attributed to a mop‐up campaign to test the children of index patients. In DRC, EID testing rebounded after initial declines due to Stop‐Work orders. This can be partially attributed to the use of point‐of‐care diagnostic technologies that helped mitigate against disruptions in sample transportation, as well as the use of specific staff to follow up on any missed sample collection for EID among the HIV‐exposed infants.

In three of the four countries, the abrupt disruptions of services demonstrated the integral role of PEPFAR support in delivering HIV testing, treatment and PVT services, including EID. The findings suggest that even in the presence of waivers, the disruption of services remained in some countries, largely due to the prolonged stoppage of PEPFAR/USAID contracts. A study that solicited responses from 65 PEPFAR partner organizations found that, despite the waivers, most reported large disruptions in HIV services due to complexities in the reinitiation of work. They cited the slow processes for approving workplan modifications to align with waivers and the fear of expending funds without a guarantee of reimbursement. For example, 91%, 87% and 82% of the organizations indicated that HIV testing, treatment and PVT services had been either cancelled or scope reduced, respectively, even though these activities were included in the waiver [7].

## 5 CONCLUSIONS

In summary, the disruptions noted in association with the Stop‐Work orders should serve as a caution about the potential threats of a substantial reduction in foreign assistance for HIV programming. To sustain gains and prevent a resurgence of HIV globally through disruptions in treatment causing viral rebound and rise of drug resistance, careful planning and realistic timelines are necessary, as well as accounting for the country's context, state of the health system and the availability of domestic or alternative resources. Lastly, the findings highlight the urgency in identifying cost‐effective models of service delivery that allow for the provision of high‐quality programmes that meet the needs of recipients of care while addressing health system constraints.

## COMPETING INTERESTS

The authors declare no competing interests.

## AUTHOR CONTRIBUTIONS

SS, MF and WME conceptualized the study. SK, LM, AG, HAW, EP, NM, LB and TD oversaw the data collection. SS and MF analysed and visualized the data. SS, MF and WME drafted the manuscript. All authors had access to the data, contributed to the interpretation of the results, critically reviewed and edited the manuscript, and had final responsibility for the decision to submit the manuscript for publication.

## FUNDING

The funding for the HIV care and treatment programmes was funded by cooperative agreements with the CDC, including NU2GGH002502 (Angola), NU2HGH000083 (DRC), NU2GGH002496 (South Sudan) and NU2GGH002254 (Zambia).

## Data Availability

The data that support the findings of this study are available from the corresponding author upon reasonable request.
